# RGS1 and related genes as potential targets for immunotherapy in cervical cancer: computational biology and experimental validation

**DOI:** 10.1186/s12967-022-03526-0

**Published:** 2022-07-25

**Authors:** Siyang Zhang, Han Wang, Jiao Liu, Tao Tao, Zhi Zeng, Min Wang

**Affiliations:** Department of Obstetrics and Gynecology, Shengjing Hospital of China Medical University, Shenyang, China

**Keywords:** Cervical cancer, Immune checkpoint inhibitors, Computational biology, Immune infiltration

## Abstract

**Background:**

Effective treatment is needed for advanced, inoperable, or chemotherapy-resistant cervical cancer patients. Immunotherapy has become a new treatment modality for cervical cancer patients, and there is an urgent need to identify additional targets for cervical cancer immunotherapy.

**Methods:**

In this study the core gene, RGS1, which affects immune status and the FIGO stage of cervical cancer patients was identified by WGCNA analysis and differential analysis using TCGA database. 10 related genes interacting with RGS1 were identified using PPI network, and the functional and immune correlations were analyzed. Based on the expression of RGS1 and related genes, the consensus clustering method was used to divide CESC patients into two groups (group 1, high expression of RGS1; group 2, low expression of RGS1). Then, the functional enrichment analysis was used to search for the functional differences in differentially expressed genes (DEGs) between group 1 and group 2. Immune infiltration analysis was performed using ESTIMATE, CIBERSORT, and ssGSEA, and the differences in expression of immune checkpoint inhibitors (ICIs) targets were assessed between the two groups. We investigated the effect of RGS1 on the clinical relevance of CESC patients, and experimentally verified the differences in RGS1 expression between cervical cancer patient tissues and normal cervical tissues, the role of RGS1 in cell function, and the effect on tumor growth in tumor-bearing mice.

**Results:**

We found that RGS1 was associated with CD4, GNAI3, RGS2, GNAO1, GNAI2, RGS20, GNAZ, GNAI1, HLA-DRA and HLA-DRB1, especially CD4 and RGS2. Functional enrichment of DEGs was associated with T cell activation. Compared with group 2, group 1 had stronger immune infiltration and higher ICI target expression. RGS1 had higher expression in cervical cancer tissues than normal tissues, especially in HPV-E6 positive cancer tissues. In cervical cancer cell lines, knockdown of RGS1 can inhibited cell proliferation, migration, invasion, and tumor growth in nude mice and promoted apoptosis.

**Conclusions:**

RGS1, as an oncogenic gene of cervical cancer, affects the immune microenvironment of patients with cervical cancer and may be a target of immunotherapy.

## Background

Cervical cancer is a gynecologic malignancy with the highest incidence among women of childbearing age [1]. An estimated 342,000 women died of cervical cancer in 2020 [2]. Despite the availability of preventive vaccines, cervical cancer remains an important health problem in developing countries. According to the results published by the International Federation of Obstetrics and Gynecology, patients with stage IB-IIA have a 10–20% risk of recurrence, while patients with stage IIB-IVA have a 50–70% risk of recurrence. Moreover, patients with distant metastases and locally uncontrolled disease recurrence have a worse prognosis [3].

The cervical cancer FIGO stage is crucial to guide further treatment. Early-stage tumors can be treated with prior surgery, while locally advanced diseases should be treated with a combination of radiotherapy, chemotherapy, and brachytherapy [4]. Unfortunately, some cervical cancer patients are resistant to chemotherapy. Tewari et al. reported that the median progression-free survival (PFS) and overall survival (OS) of the first platinum-based chemotherapy occur at 6 and 12 months, respectively [5]. These results were clearly disappointing. One promising approach to treat these chemotherapy-resistant cervical cancer patients is immunotherapy. Enhancing the host immune system can facilitate anti-tumor immune surveillance. In several trials involving patients with recurrent cervical, vaginal, and vulvar cancers, anti-PD1 and PD-L1 antibodies have been shown to significantly shrink tumors in 15–20% of patients [6]. The use of ICIs has greatly changed the therapeutic pattern for many solid organ malignancies. An ever-increasing number of targets have been identified, and relevant studies have made people have provided better insight into the underlying mechanism and efficacy of ICIs in cervical cancer treatment [7].

Regulators of G-protein signaling (RGS) proteins were originally discovered because they enhance the intrinsic GTPase activity of heterotrimer G proteins, acting as inhibitors of G protein-coupled receptor (GPCR) activated signal pathways [8]. RGS1, as the most important member of the RGS family, mainly exists in hematopoietic cells, including T lymphocytes, B lymphocytes, natural killer cells, dendritic cells, and monocytes [9, 10]. Several studies have demonstrated that high expression of RGS1 inhibits the chemotaxis of immune cells [11, 12]. In recent years, RGS1 has been mainly studied in tumors, especially in melanomas. RGS1 promotes melanoma progression by regulating Gαs-mediated inactivation of AKT and ERK, and is a novel therapeutic target and prognostic marker for melanomas [13, 14]. In addition, RGS1 is highly expressed in multiple malignancies and predicts poor prognosis in cancers [15].

The cervical cancer transcriptome data from The Cancer Genome Atlas (TCGA) were analyzed to find genes that may be targets for cervical cancer immunotherapy. Based on WGCNA analysis and FIGO stage differential gene analysis, we identified the core gene RGS1 and 10 related key genes. The patients were divided into two groups according to the levels of genes expressions, and bioinformatics methods were used to analyze the differences in biological function, immune cell infiltration, immune checkpoint expression and prognosis.

## Methods

### Data sources and pre-processing

The dataset of 296 cervical cancer patients was downloaded from TCGA using the TCGAbiolinksR package [16]. This dataset includes normalized RNA expression data and clinical characteristics (age, tumor type, FIGO stage, etc.). Tumor tissue samples were further analyzed by ${\text{log}}_{{2}} \left( {\text{FPKM}} + 1 \right)$ transformation, and differences were analyzed by HTSeq-Counts.

### WGCNA reveals key modules and hub genes associated with CESC immunity

WGCNA algorithm was used to analyze the 5000 genes with the largest variance in TCGA, and showed the strongest immune-related modules and genes [17]. First, in order to make the network structure conform to the approximate scale-free topology criterion, it was necessary to select the soft threshold power. Second, network adjacencies were determined and transformed into the topological overlap matrix (TOM), and hierarchical clustering was performed according to the similar expression characteristics of genes. Third, to ensure that each module had at least 20 genes, the modules were distinguished using the dynamic tree-cutting algorithm [18]. Modules with high similarity were merged. After constructing the network, the immune-related modules and genes were determined by calculating the module-trait correlations, gene significance (GS, correlation coefficient between genes and traits), and module membership (MM, correlation coefficient between genes and module eigengenes). Ultimately, we obtained one immune-related module and 270 hub genes.

### DEGs between FIGO stage I & II and stage III & IV

Expression analysis data (HTSeq-Counts) was compared to identify DEGs between FIGO stage I & II and stage III & IV using R package DESeq2 [19]. The DEGs were filtered with the thresholds $\left| {{\text{log}}_{{2}} {\text{FoldChange}}} \right| < 1$ and $P{\text{ value}} < 0.05$. We intersected the DEGs with genes of WGCNA to obtain a core gene.

### Functional enrichment analysis

We used PPI networks to identify protein partners that interact with the core gene in the STRING database [20]. We obtained 11 interacting key genes, including the core gene. Gene Ontology (GO) analysis was performed using the R package clusterProfiler to understand the function of 11 genes [21]. Next, we used R package corrplot to analyze the Spearman’s correlation between gene and gene, the correlation between gene and ESTIMATE, the correlation between gene and SSGSEA.

### Immune infiltration analysis

The stromal to immune cells ratio in tumor samples was determined using an ESTIMATE analysis. The tumor microenvironment (TME) of CESC patients was assessed using the R package estimation method, including StromalScore (stromal content), ImmuneScore (degree of immune cell infiltration), ESTIMATEScore (synthetic marks for stroma and immune), and TumorPurity [22]. CIBERSORT is a method for calculating cell composition according to expression profiles, which was used to determine the ratio of 22 immune cells per CESC patient [23]. The 28 published immune cell types were calculated using single-sample Gene Set Enrichment Analysis (ssGSEA) in the R package GSVA [24].

### Consensus clustering of the key genes

We used the R package ConsensusClusterPlus to divide CESC patients into two groups to further clarify the biological characteristics and value in immune infiltration of key genes [25]. DEGs in the two groups were identified by the R package limma [26].

### Gene set enrichment analysis

GO, Kyoto Encyclopedia of Genes and Genomes (KEGG) and Gene Set Enrichment Analysis (GSEA) were used to analyze the biological functions of the DEGs [21]. GO analysis mainly consists of three complementary biological concepts: Biological Process (BP), Molecular Function (MF) and Cellular Component (CC). Furthermore, KEGG integrates genomic, chemical and system function information to understand the advanced and practical functions of biological systems such as normal and cancer cells. GSEA is a calculation method that analyzes the enrichment of preset sets in specific rankings [27]. In this study, we utilized this algorithm to analyze the known signaling pathways associated with DEGs.

### qRT-PCR

Total RNA was extracted from cells using the TRIzol reagent (16,096,020, Invitrogen, USA). mRNA was reverse transcribed into cDNA using the PrimeScript™ RT Reagent Kit with gDNA Eraser (RR047A, Takara, USA). TB Green Premix Ex Taq II (RR820A, Takara, USA) and cDNA were added to the 20 µL system. The real-time quantitative PCR was performed with QuantStudio (A40425, Thermo, USA). The RGS1 primer sequences were 5′-AGTCTGATCTTTTGCCCTGTAA-3′ and 3′-TTAGCCTGCAGGTCATTTAGAA-5′. The mRNA expression level was normalized to the GAPDH expression level, and the relative quantification was expressed by 2^−∆∆ct^. All experiments were repeated three times.

### Immunohistochemistry and image analysis

The fresh tissues were fixed, embedded, and sectioned (4 μm thick). The tissue sections were analyzed using the immunohistochemistry kit (E-IR-R211, Elabscience, China). Slides were incubated with RGS1 Polyclonal Antibody (E-AB-11534, Elabscience, China) and HPV16 + 18-E6 Monoclonal Antibody (ab70, abcam, USA) at 4 °C overnight. Slides were photographed under a microscope (703,548, Nikon, Japan).

### Cell culture and shRNA transfection

HeLa (CL-0101, Procell, China) and SiHa (CL-0210, Procell, China) were cultured in RPMI 1640 (SH30255.01, HyClone, USA) with 10% fetal bovine serum (FBS) (164,210, Procell, China) at 37 °C and 5% CO_2_. The sequence of the RGS1 gene (GeneID: 5996) was obtained from the National Biotechnology Information Center (NCBI). sh-RGS1 (5′-UGAAUGAGUGGUUCCUUUCAA-3′ and 3′-GAAAGGAACCACUCAUUCACU-5′) and sh-NC plasmids were transfected into cervical cancer cells, respectively.

### Western blot

A mixture with 37.5 µg of protein and 5× loading buffer was added to 10% SDS–polyacrylamide gel for electrophoresis and transferred to polyvinylidene fluoride (PVDF) membrane (BS-PVDF-22, Bioshop, China). After sealing, the cells were incubated overnight with RGS1 Polyclonal Antibody (E-AB-11534, Elabscience, China) at 4 ℃. The membrane was coupled with goat anti-rabbit IgG antibody (1:5000, 31,466, Invitrogen, USA) at room temperature for one hour. The relative protein content was detected using ImageJ software density method. Rabbit anti-GAPDH polyclonal antibody (1:2500, AB9485, ABCAM, USA) was used as the loading control.

### CCK8

The collected cells from each group were added to 96-well plates at a density of 2000 cells/well and cultured for 0, 24, 48, 72, and 96 h. Five parallel wells were used in the sh-RGS1 and sh-NC groups. 10 µL CCK-8 (HB-CCK8-2, Hanbio, China) reagent was added to each well. Absorbance was measured at 450 nm using a microplate meter after 2 h (1603301D, Bio Tek, USA).

### Transwell

For Transwell chamber (3413, Corning, USA), 200 µL serum-free medium containing 5*10^4^ cells were added to the upper chamber and 800 µL serum-containing medium was added to the lower chamber for incubation for 2 days. The staining chamber was fixed and photographed using the inverted microscope. The number of submembrane cells was calculated using Image-Pro Plus 6.0. The cell invasion assay required the addition of 10 µL Matrigel on the Transwell chamber (356,234, BD, USA; 1/5 dilution RPMI 1640).

### Apoptosis assay

The cells of each group were resuspended in 500 µL buffer after twice-washing in pre-cooled PBS. The apoptosis detection kit (CA1020, Solarbio, China) was used to detect cell apoptosis. Annexin V-FITC fluorescein isothiocyanate (FITC) was added and incubated for 30 min before adding PI dye. The cell apoptosis rate was measured by flow cytometry after 15 min.

### Immunofluorescence

To detect the localization of RGS1 protein, HeLa cells were immobilized and permeated. After cells and anti-human antibodies (E-AB-11534, Elabscience, China) were incubated overnight at room temperature, immunofluorescence staining was performed with Rabbit/Mouse IgG-H&L (1:200, ab150079/ ab150115, abcam, USA) and 4ʹ, 6-diamino-2-phenylindoles (DAPI) (S2110, Solarbio, China). The images were obtained using a confocal microscope.

### Tumor growth studies

Transfected cells (2*10^6^) were injected subcutaneously into the back of female Balb/c nude mice. The tumor volume (0.5*a^2^*b) and weight of the tumor were recorded.

### Statistics analysis

R software (4.0.2) was used to perform all statistical analyses. GraphPad Prism 8.0 version was also applied for data analysis. Box plot analysis was performed using the Wilcoxon rank sum test. Spearman’s coefficient was used for correlation analysis. All hypothesis tests were bilateral and $P < 0.05$ was considered the statistically significant difference.

## Results

### WGCNA and differential expression analysis were used to search for immune core genes affecting FIGO stage

**Fig. 1:**
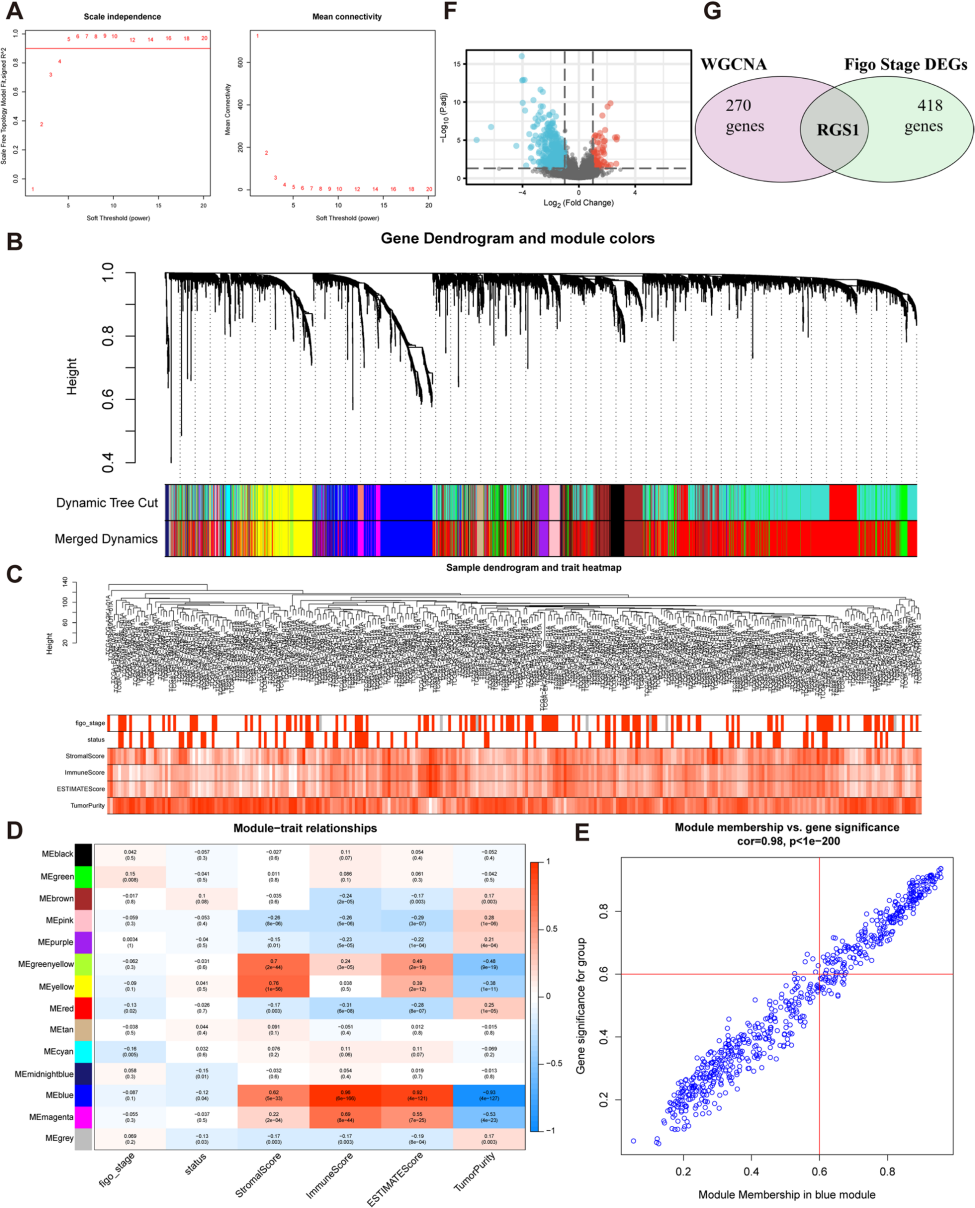
Search for the core gene RGS1 related to cervical cancer immunity and FIGO Stage. **A** Determine that the soft threshold for network topology analysis is 5. **B** Generate gene dendrogram and color modules. **C** Sample clustering heatmap. **D** Heatmap between module characteristic genes and ESTIMATE results. **E** Scatter plot of genes in the blue module. **F** DEGs between FIGO Stage I & II and Stage III & IV. **G** The core gene-RGS1 was obtained by intersection of DEGs and WGCNA results

WGCNA algorithm was used to analyze genes in the top 5000 of variance in TCGA. After selecting appropriate soft powers (Fig. 1A) and clustering parameters, 5000 genes were divided into 14 modules (Fig. 1B). The ESTIMATEScore of all the samples were showed (Fig. 1C). According to the results of the correlation heatmap, we selected the blue module with the highest correlation with the ImmuneScore (R = 0.96, P = 6e−166) (Fig. 1D). Thereafter, according to MM > 0.6 and GS > 0.6 conditions, 270 HUB genes were obtained in the blue module (Fig. 1E). We intersected these 270 HUB genes with DEGs of FIGO stage I & II and stage III & IV (Fig. 1F) to obtain our core gene—RGS1 (Fig. 1G).

### Functional enrichment and correlation analysis of key genes

**Fig. 2:**
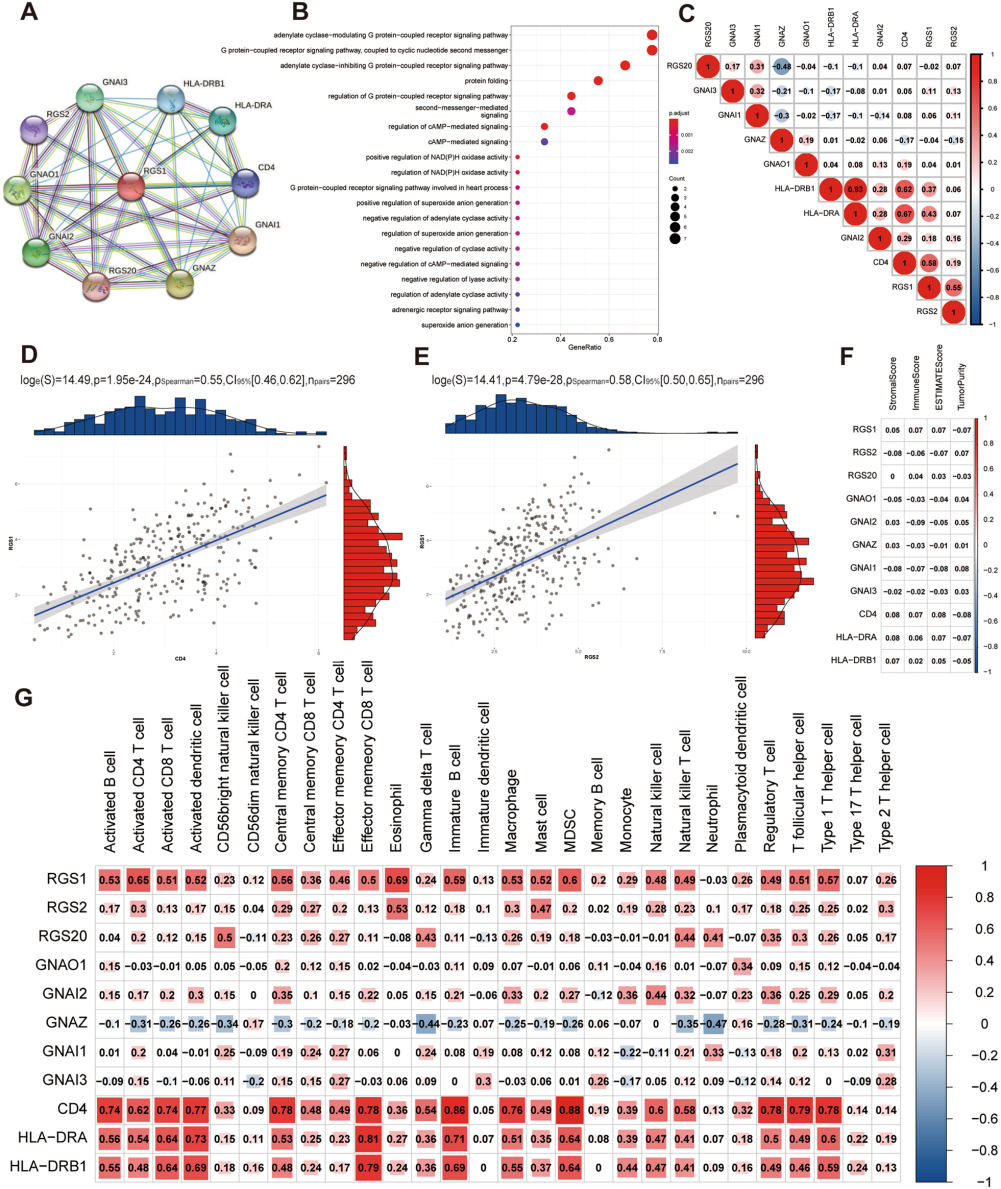
Functional enrichment and correlation analysis of key genes. **A** PPI analysis of genes interacting with RGS1. **B** GO enrichment analysis of 11 key genes. **C** Gene–gene correlation analysis. **D, E** Correlation of RGS1 with CD4 and RGS2. **F** Correlation between key genes and ESTIMATE analysis. **G** Correlation between key genes and expression of immune cells (ssGSEA)

We performed PPI network and found 10 genes associated with RGS1 protein function. We designated these 10 genes as key genes along with RGS1 (Fig. 2A). GO enrichment analysis of 11 key genes showed that most of these genes were enriched in the adenylate cyclase-modulating GPCR signaling pathway and affected protein folding (Fig. 2B). In the exploration of gene–gene correlation, we showed that RGS1 was strongly correlated with CD4 and RGS2 (Fig. 2C). Therefore, the Spearman correlation between RGS1 and CD4 and RGS2 was studied. It was found that RGS1 was positively correlated with CD4 (R = 0.58, P = 4.79e−28) (Fig. 2D) and RGS2 (R = 0.55, P = 1.95e−24) (Fig. 2E). The Spearman correlation analysis of genes associated with immune infiltration (ESTIMATE and ssGSEA) results showed that RGS1, CD4, HLA-DRA, and HLA-DRB1 were significantly associated with immune infiltration (Fig. 2F, G).

### Consensus clustering of CESC patients and functional enrichment analysis of DEGs

**Fig. 3:**
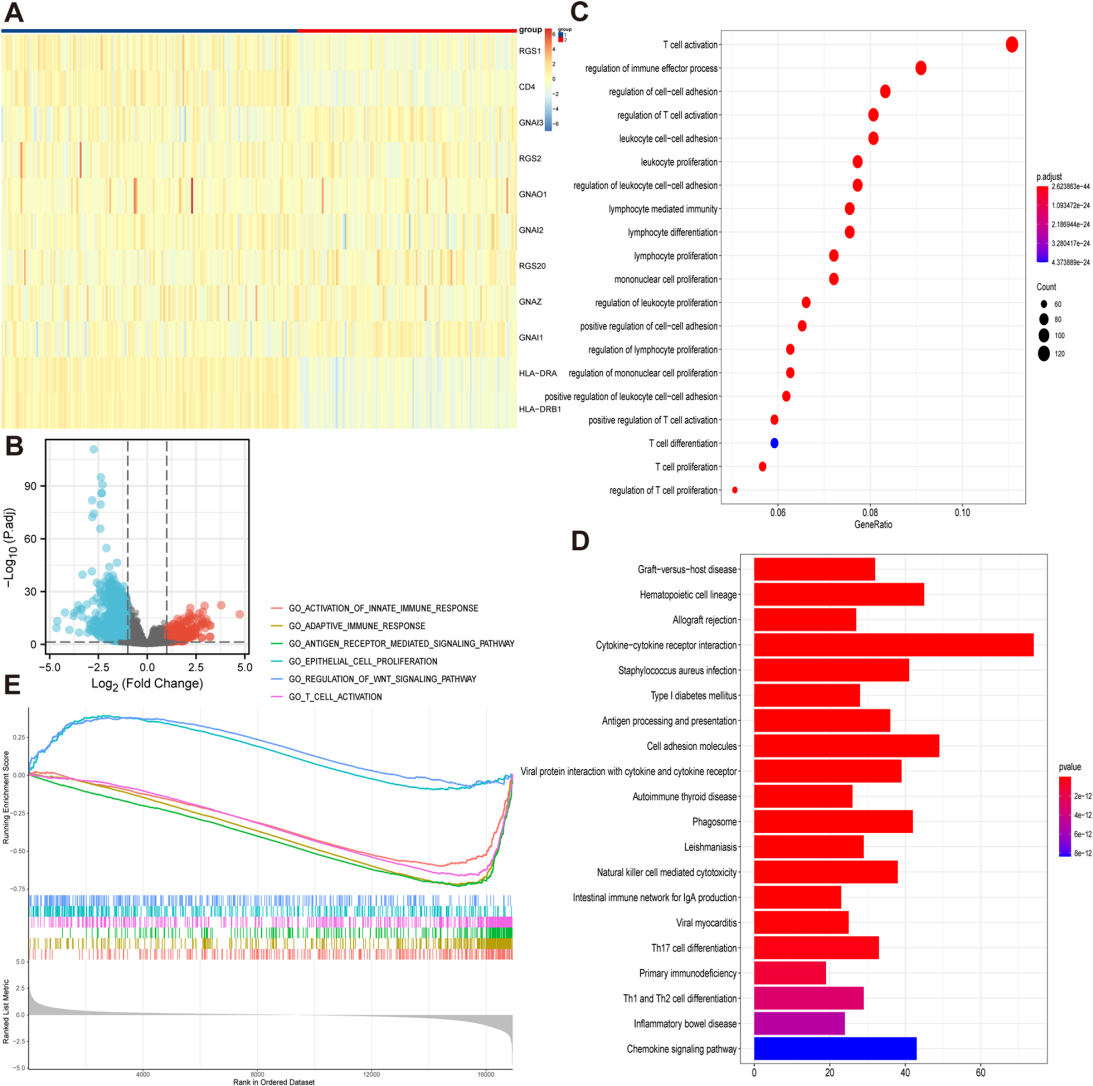
Consensus clustering of CESC patients and functional enrichment analysis of DEGs. **A** CESC patients are divided into two groups according to expression of key genes. **B** Volcano plot of DEGs. **C** The GO analysis of key genes. **D** The KEGG analysis of key genes. **E** The GSEA analysis of key genes

To determine whether the key genes in patients affected immune function, we performed consistent clustering of 296 CESC samples according to the key genes expression matrix and divided the samples into two groups (Fig. 3A). We analyzed the DEGs between the two groups and obtained 1361 DEGs (Fig. 3B). These genes were used for functional and pathway enrichment analyses to determine the underlying mechanisms responsible for the differences between the two groups. “T cell activation”, “regulation of immune effector process”, and “regulation of cell–cell adhesion” were the most common GO terms (*P* < 0.001) (Fig. 3C). In KEGG analysis, “cytokine-cytokine receptor interaction”, “cell adhesion molecules” and “chemokine signaling pathway” were the top three enrichment pathways (*P* < 0.001) (Fig. 3D). The GSEA enrichment of MSigDB Collection (c5.cp.v7.0.symbols.gmt) identified numerous important pathways related to immune and tumor development, including activation of the innate immune response, adaptive immune response, antigen receptor mediated signaling pathway, epithelial cell proliferation, regulation of WNT signaling pathway and T cell activation (Fig. 3E).

### Comparison of immune infiltration

**Fig. 4:**
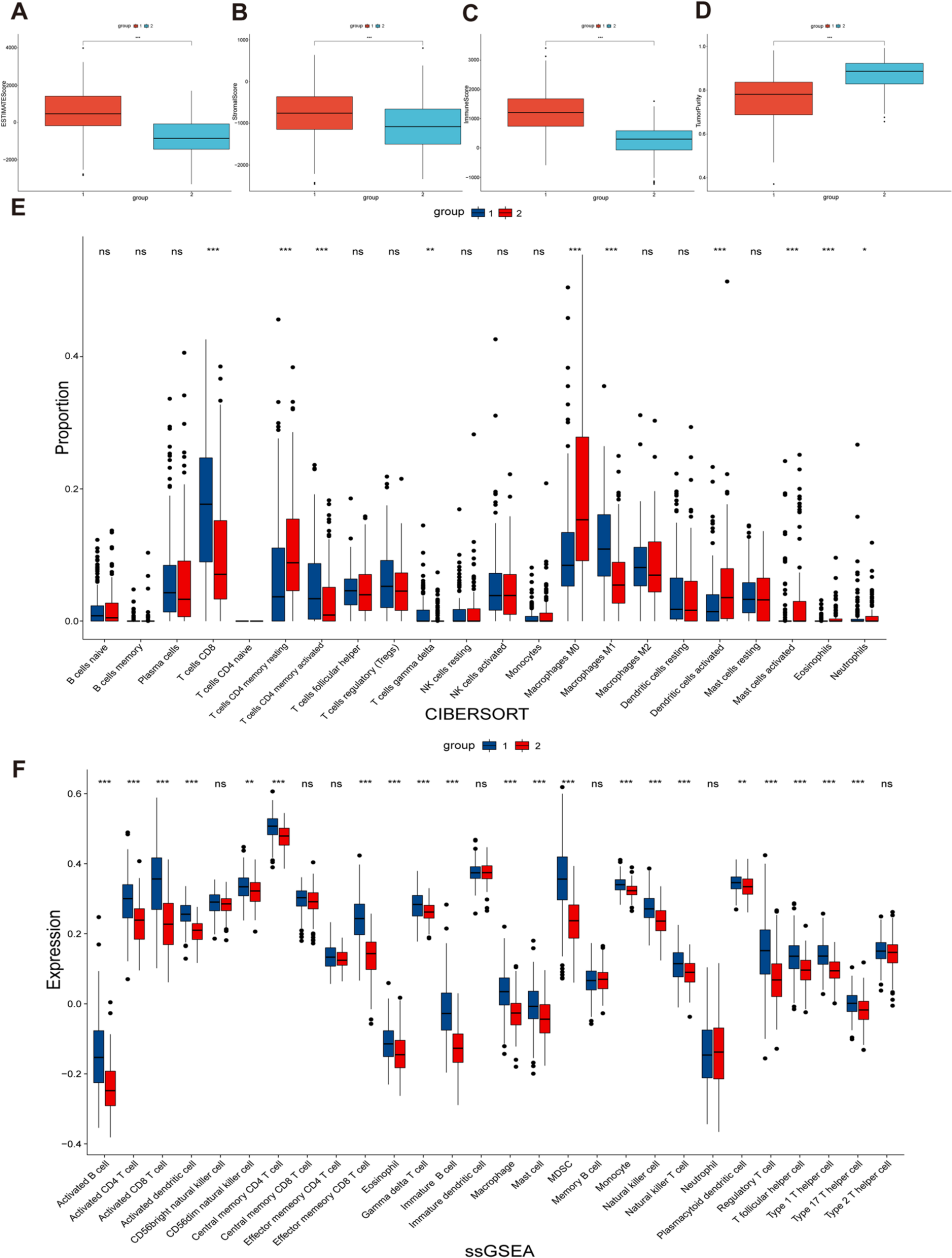
Comparison of immune infiltration. **A** Comparison of StromalScore between two groups. **B** Comparison of ImmuneScore between two groups. **C** Comparison of ESTIMATEScores between two groups. **D** Comparison of TumorPurity between two groups. **E** Comparison of proportion of immune cells between two groups. **F** Comparison of expression of immune cells between two groups

To better characterize the differences in immune function between the two groups, we performed ESTIMATE, CIBERSORT, and ssGSEA analyses. ESTIMATE analysis confirmed lower StromalScore (Fig. 4A), ImmuneScore (Fig. 4B), and ESTIMATEScore (Fig. 4C) and higher TumorPurity (Fig. 4D) in group 2 than in group 1. In addition, CIBERSORT analysis showed that group 2 had a higher percentage of CD8 T cells and activated CD4 memory T cells (Fig. 4E). The similar conclusions were drawn from ssGSEA analysis. (Fig. 4F).

### Evaluation of sensitivity of immune target therapy

**Fig. 5:**
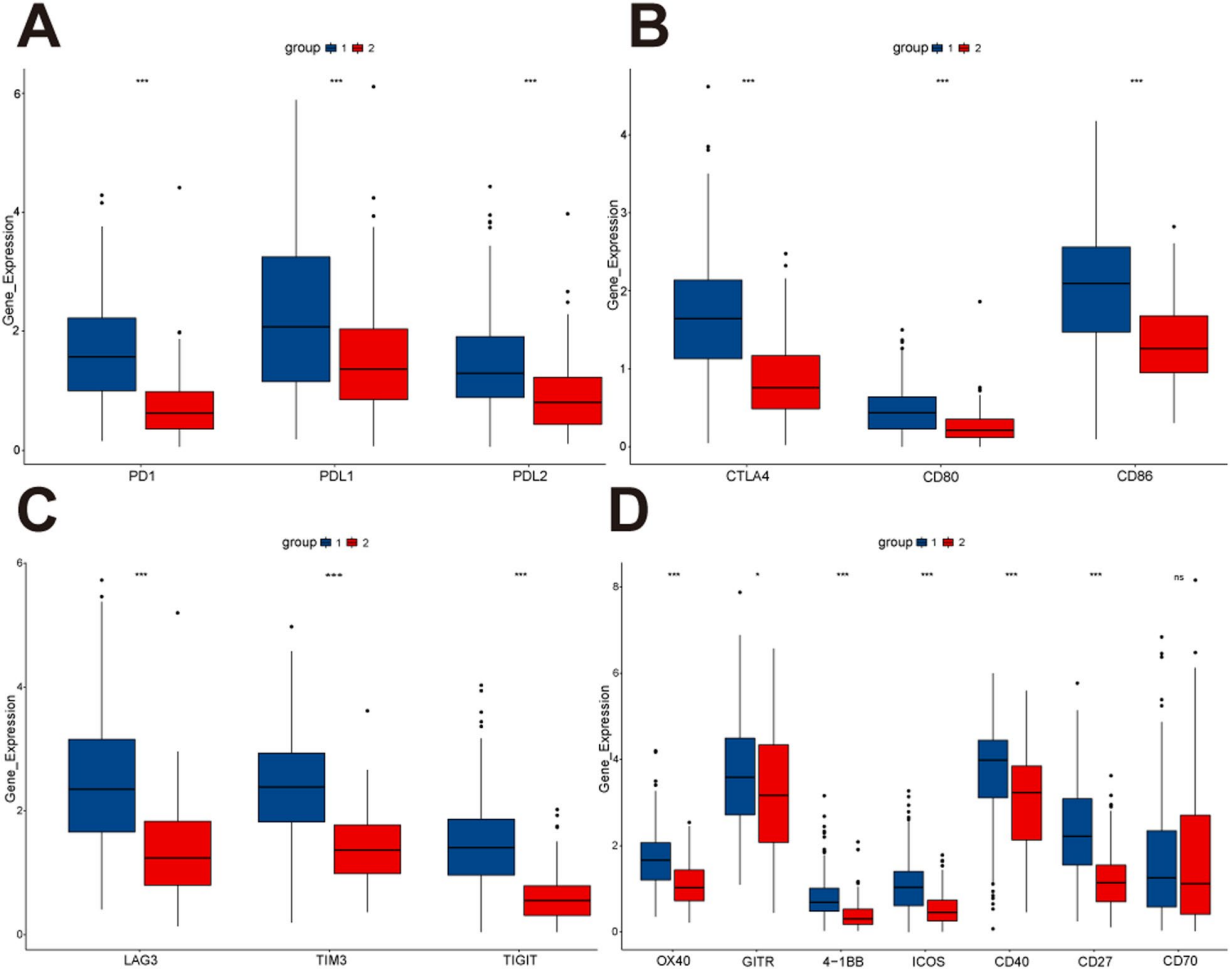
Comparison of the expression levels of immunomodulatory drug clinical trial targets in cervical cancer between two groups. **A** The expression levels of PD1, PD-L1 and PD-L2 between two groups. **B** The expression levels of CTLA-4, CD80 and CD86 between two groups. **C** The expression levels of LAG3, TIM3 and TIGIT between two groups. **D** The expression levels of OX40, GITR, 4-1BB, ICOS, CD40 CD27 and CD70 between two groups

To assess the sensitivity of two clusters of CESC patients to immune target therapy, we compared the expression of immunoregulatory targets commonly used in clinical trials between the two groups. The results suggested that group 1 had a better response to immunomodulatory targets (PD1, PD-L1, PD-L2, CTLA-4, CD80, CD86, LAG3, TIM3, TIGIT, OX40, GITR, 4-1BB, ICOS, CD40, and CD27) than group 2 (Fig. 5A–D).

### Clinical relevance of RGS1

**Fig. 6:**
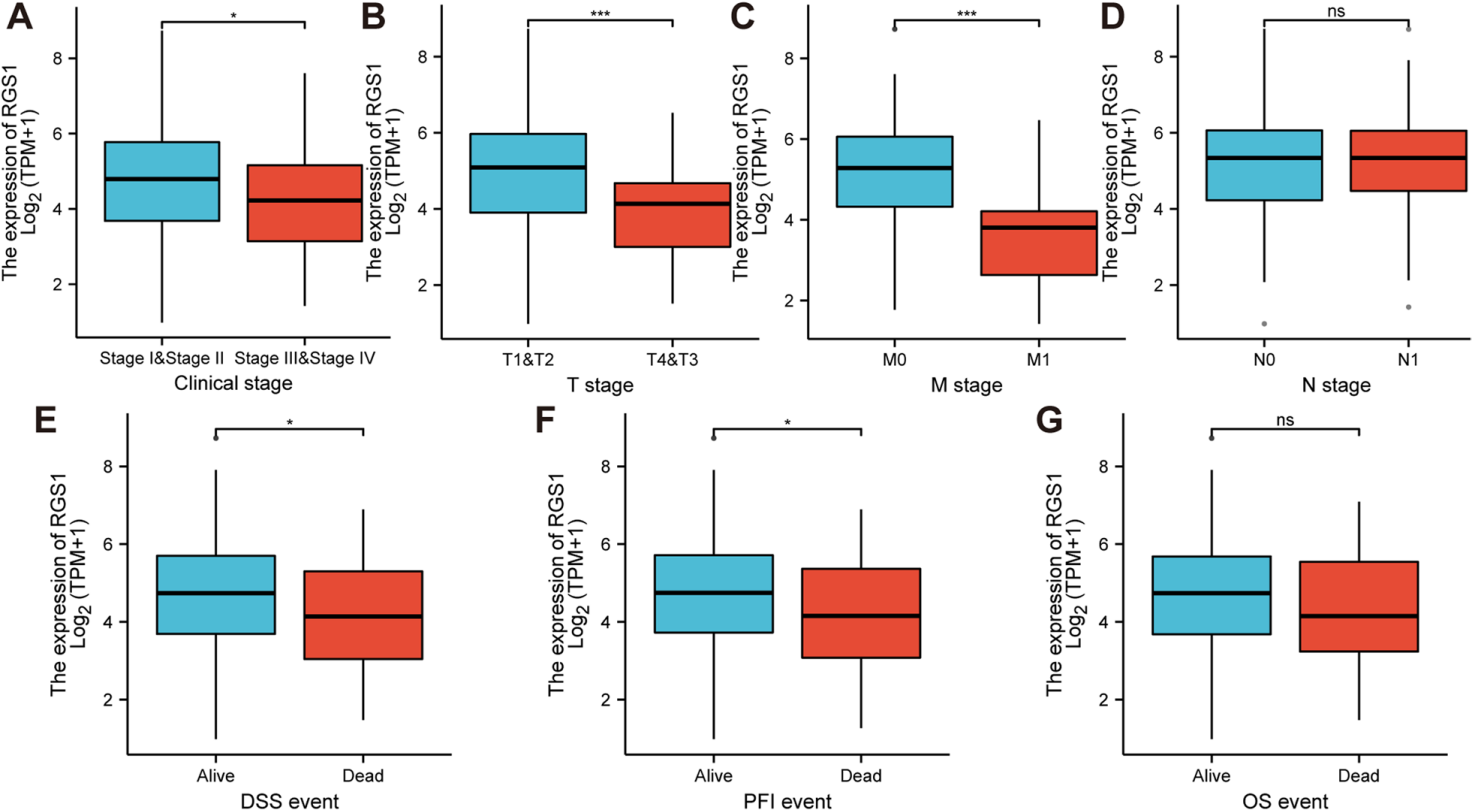
Clinical relevance of RGS1. **A** FIGO stage. **B** T stage. **C** M stage. **D** N stage. **E** DSS event. **F** PFI event. **G** OS event

We focused on the effect of RGS1 on the clinical prognosis of the CESC patients. We found that the high expression of RGS1 inhibited the progression to FIGO stage III & IV in CESC patients (Fig. 6A). With respect to TNM stage, the level of RGS1 expression in T3&T4 stage was lower than T1&T2 stage (Fig. 6B). The level of RGS1 expression in the CESC patients at M1 stage was lower than patients at M0 stage (Fig. 6C). However, the level of RGS1 expression had no effect on N stage (Fig. 6D). In an analysis of disease specific survival (DSS) and progress free interval (PFI) in CESC patients, low expression of RGS1 may lead to patient death (Fig. 6E, F). However, there was no significant difference in RGS1 expression in OS (Fig. 6G).

### Levels of RGS1 expression verification in tissues and cells

**Fig. 7:**
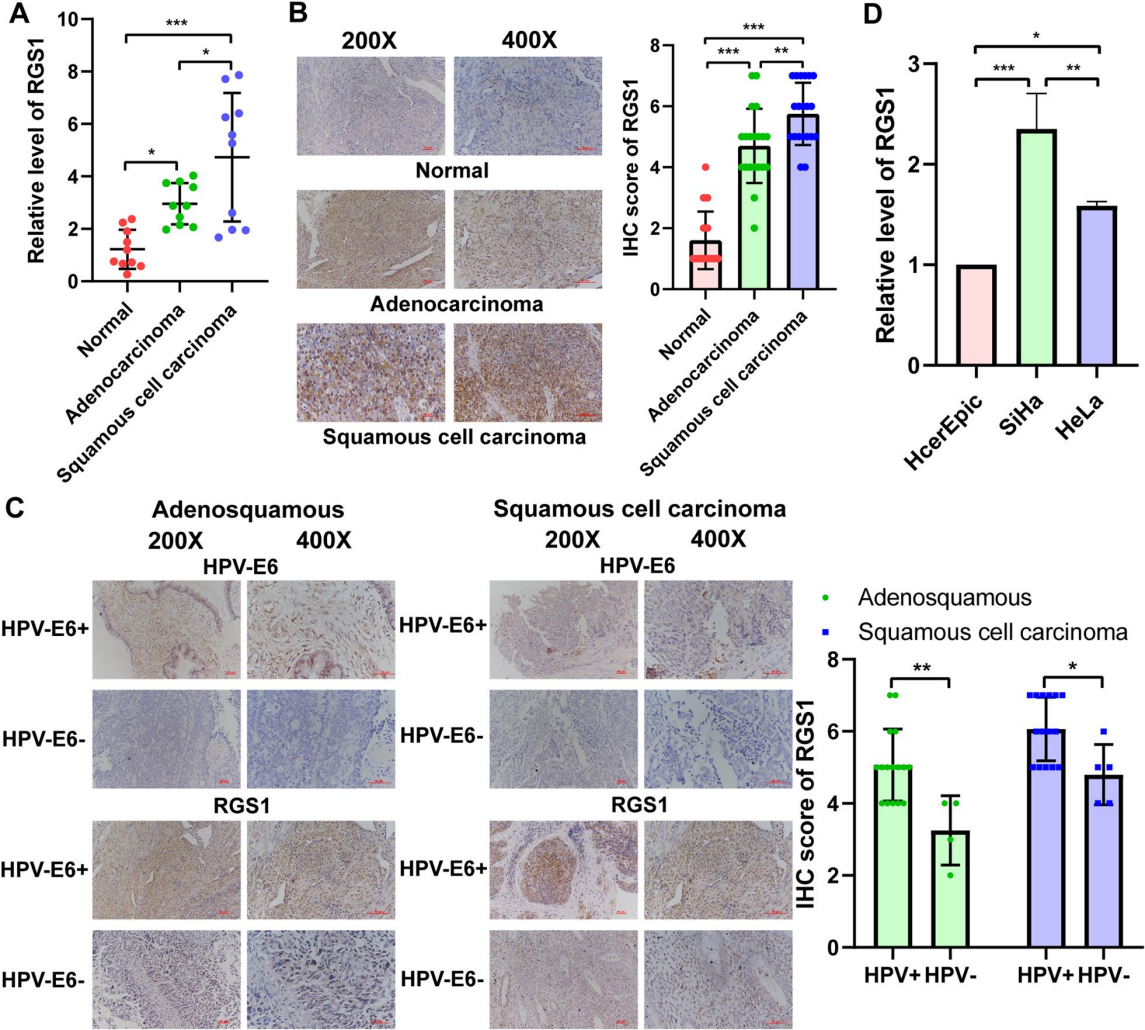
Verification of expression levels of RGS1 in tissues and cells. **A** The expression of RGS1 was detected by qRT-PCR in tissues. **B** The expression of RGS1 was detected by immunohistochemistry in tissues. **C** The expression of RGS1 was detected by immunohistochemistry between HPV-E6-positive cancer tissues and HPV-E6-negative cancer tissues. **D** The expression of RGS1 was detected by qRT-PCR in cells

First, we detected RGS1 mRNA expression in 10 cervical adenocarcinoma carcinoma tissues, 10 cervical squamous cell carcinoma tissues, and 10 normal cervical tissues. We found that the quantity of RGS1 mRNA expression in cervical carcinoma tissues was higher than normal cervical tissues, and the quantity of RGS1 mRNA expression in squamous cell carcinoma tissues was increased compared with adenocarcinoma carcinoma tissues (Fig. 7A). Next, we determined the level of RGS1 protein expression in 20 cervical adenocarcinoma carcinoma tissues, 20 cervical squamous cell carcinoma tissues, and 20 normal cervical tissues by immunohistochemistry. Similarly, compared with normal cervical tissues, the quantity of RGS1 protein expression was increased in cervical carcinoma tissues and the quantity of RGS1 protein expression in squamous cell carcinoma tissues was higher than adenocarcinoma carcinoma tissues (Fig. 7B). Further exploration found that the RGS1 protein expression was higher in HPV-E6-positive adenocarcinoma and squamous cell carcinoma tissues than HPV-E6-negative carcinoma tissues (Fig. 7C). Moreover, qRT-PCR confirmed that the expression of RGS1 in the HeLa (adenocarcinoma) and SiHa cell lines (squamous cell carcinoma) was higher than the HcerEpic cell line (normal epithelial cells of the cervix) (Fig. 7D).

### RGS1 promotes cervical cancer development in vivo and in vitro

**Fig. 8:**
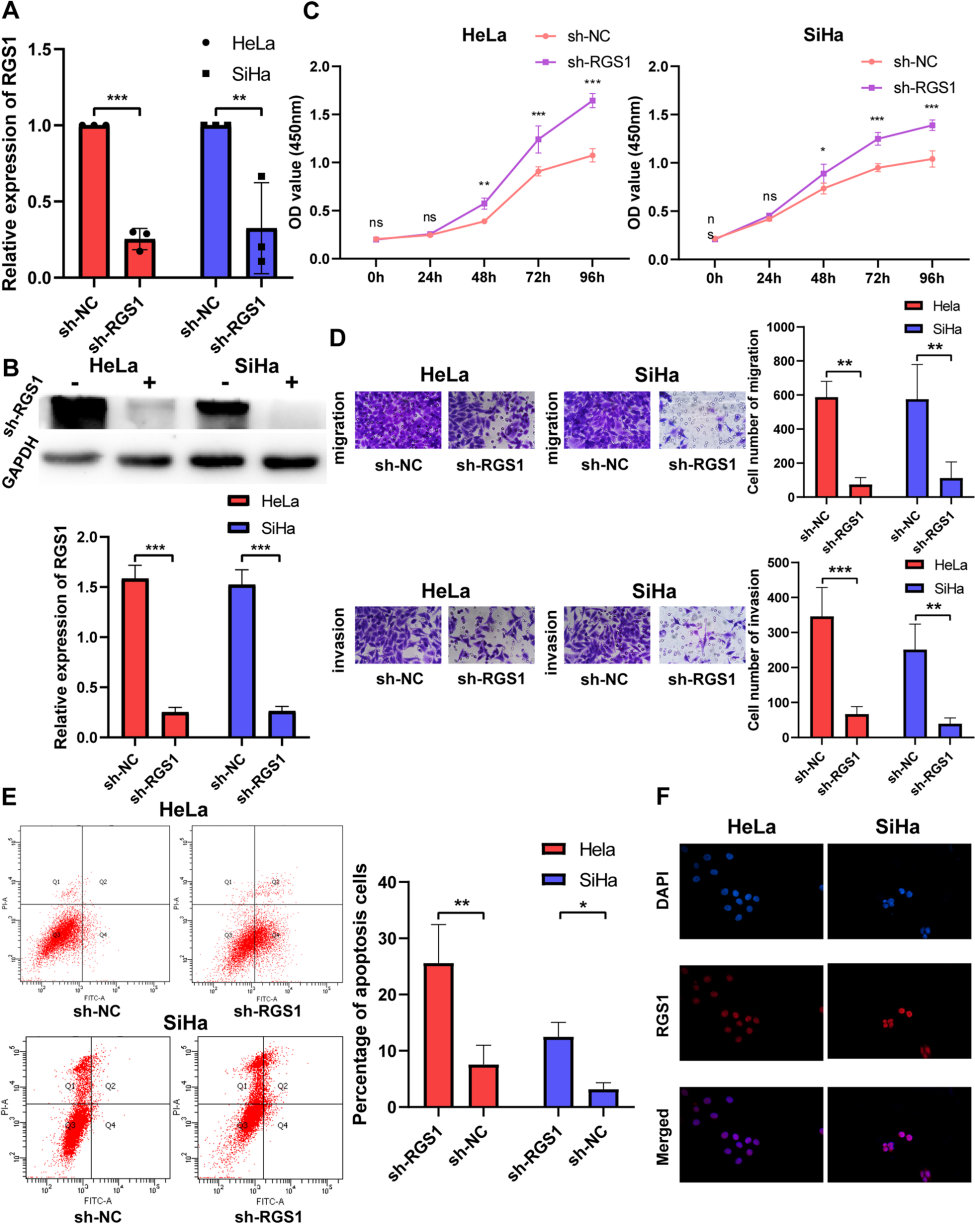
Effect of RGS1 knockdown on cervical cancer cells. **A** qRT-PCR was used to detect the expression of RGS1 after knockdown of RGS1 in HeLa and SiHa cells. **B** Western Blot was used to detect the expression of RGS1 after knockdown of RGS1 in HeLa and SiHa cells. **C** CCK8 detected the effect of RGS1 on cell proliferation. **D** Transwell examined the effects of RGS1 on cell migration and invasion. **E** FCM was used to detect the effect of RGS1 on apoptosis. **F** The localization of RGS1 in cells was determined by immunofluorescence

**Fig. 9:**
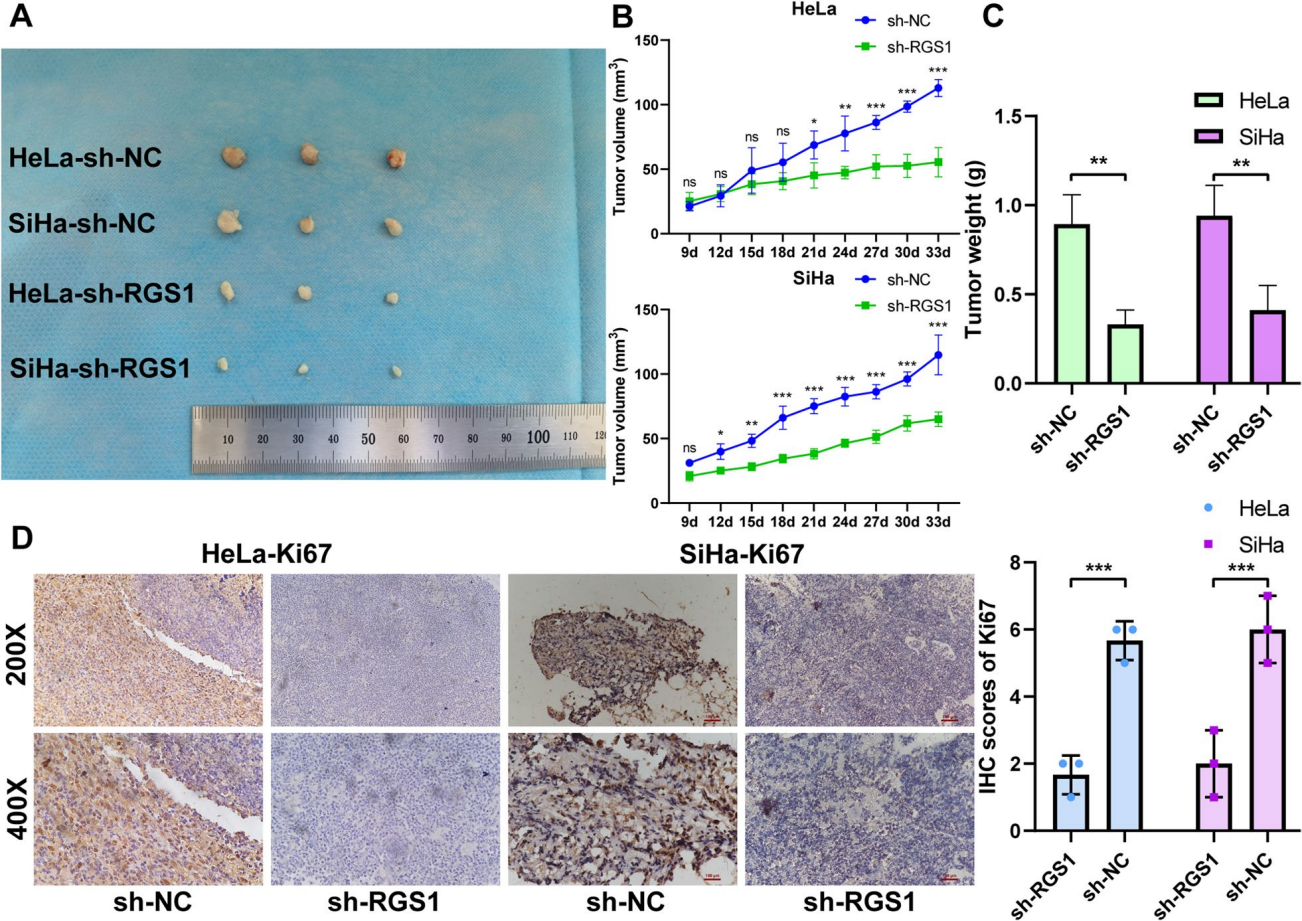
The effect of RGS1 on the growth of cervical cancer was detected by nude mice. **A** The photograph of the tumor. **B** Tumor growth curve. **C** Comparison of tumor weight between sh-RGS1 group and sh-NC group. **D** Comparison of Ki67 expression between sh-RGS1 group and sh-NC group

To study the biological functions of RGS1 in vitro, we constructed sh-RGS1 lines and control sh-NC stable cell lines. Then, qRT-PCR and Western Blot confirmed that the knockout efficiency of sh-RGS1 was 67.6–84.2% (Fig. 8A, B). CCK8 results showed that the sh-RGS1 group cell viability was significantly lower than the sh-NC group from 48 h (Fig. 8C). Transwell and flow cytometry results showed that compared with the sh-NC group, the invasion and migration of cervical cancer cells were decreased, and the apoptosis rate was increased in the sh-RGS1 group (Fig. 8D, E). Based on immunofluorescence experiments, we found that RGS1 mainly functions in the nucleus (Fig. 8F). In tumorigenesis experiments in nude mice, we found that tumor growth was slower in the sh-RGS1 group (Fig. 9A, B). After 33 days, we sacrificed all the nude mice and extirpated the tumor tissues. The tumor weight in the sh-RGS1 group was only 37% and 43.6% of the tumor weight in the sh-NC group (Fig. 9C). The Ki67 expression quantity in mouse tumor tissues was then determined by immunohistochemistry. Compared with the sh-NC group, the Ki67 expression quantity was decreased in the sh-RGS1 group (Fig. 9D).

## Discussion

**Fig. 10:**
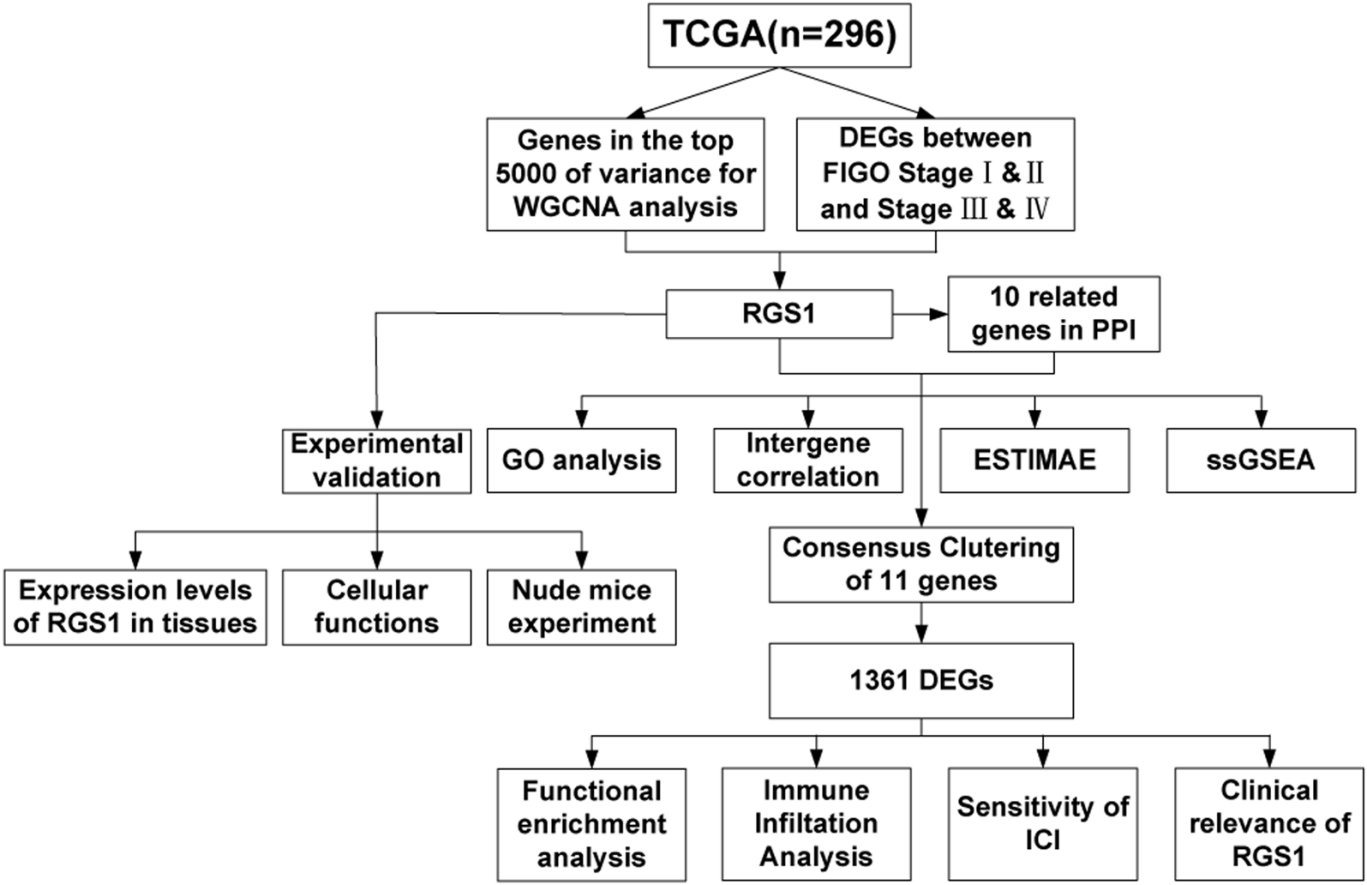
Summary of the research

In 1987, the cytotoxic T lymphocyte-associated protein 4 (CTLA-4) was discovered as the first immune checkpoint, thus opening the door to cancer immunotherapy [28]. Currently, the identified immune checkpoints have been identified: CTLA-4, PD-1 and PD-L1, LAG-3, TIM3, IDO and VISTA [29]. In a clinical trial involving 155 women with advanced cervical cancer, the objective response rate (ORR) of dual PD-1/ CTLA-4 blockade therapy was only 25.6% [30]. Therefore, an urgent need exists to find an effective immunotherapy target for the advanced cervical cancer treatment. The summary of our research is shown in Fig. 10.

We found immune-related blue modules and 270 immune-related genes by WGCNA analysis, and the core gene RGS1 was obtained by intersection of these genes with 418 differential genes based on FIGO stage. RGS1 is highly expressed in tumors. Knockout of RGS1 notably increases the invasion and survival of helper Th1 cells and cytotoxic T lymphocytes (CTLs) in breast and lung cancer transplanted tumors, and effectively inhibits tumor growth in vivo, suggesting that RGS1 is a tumor promoter [31]. RGS1 has a strong correlation with the immune microenvironment of tumors, and in helper Th1 cells and CTL, upregulation of RGS1 reduces their transport and survival in tumors. Type II interferon (IFN) and STAT1 signaling increase RGS1 expression and prevent T cell transport to tumor by inhibiting calcium influx and inactivating kinases ERK and AKT [32]. In melanoma cells, RGS1 can also be regulated by lncRNA taurine upregulated 1 (TUG1)/miR-29c-3p to promote the proliferation and invasion and inhibit cell apoptosis [33]. The role of RGS1 in cervical cancer was first revealed in the study conducted by Wong et al. in 2005. In their oligonucleotide microarray study of cancer tissues from 19 patients with early stage and 10 patients with advanced stage, they found that RGS1 expression is elevated in patients with advanced stage cancer, in contrast to our TCGA database analysis [34]. We speculate that this antithetical result may be due to the inability of early oligonucleotide microarray techniques to separate tumor and immune cells from TME as single-cell RNA sequencing does today. However, RGS1 is a real oncogenic factor in tumor cells and is known to promote immune cell maturation.

To identify genes that interact or affect RGS1, we used the STRING database and showed that 10 genes were closely associated with RGS1–RGS2, RGS20, GNAO1, GNAI2, GNAZ, GNAI1, GNAI3, HLA-DRA, HLA-DRB1. CD4 and RGS2 were the two genes most strongly associated with RGS1. In 2005, Agenès et al. found that CD4 + T cell subsets are characteristically regulated by the RGS family of genes. Regulatory T cells migrate less due to higher RGS1 expression compared to CD4 + naïve T cells, suggesting that RGS1 expression is positively correlated with maturation of CD4 + T subsets and negatively correlated with migration ability [11]. RGS2 is a signal molecule downstream of GPCR and acts as GTPase-activating protein (GAP) for several Gα subunits of G protein [35]. Kimberly et al. found that RGS2 was significantly increased in tumor-derived Myeloid derived suppressor cells (MDSCs) compared with non-tumor-derived MDSCs. Deletion of the RGS2 gene in mice significantly inhibited tumor growth and decreased tumor vascular density. These results suggest that RGS2 is involved in promoting tumor function in MDSCs [36]. We noticed that the top two pathways enriched by these 11 key genes in GO analysis were related to GPCRs signaling pathway. GPCRs play important roles in mediating cell proliferation, migration and invasion, angiogenesis, cell death, and cell survival. GPCRs also play key roles in the growth and development of cancer [37]. In HPV-associated cancers such as cervical cancer, GPCRs can be activated by the interaction of chemokines CXCR4 and ACKR3 with glycosaminoglycans of the extracellular matrix (ECM), acting as targets of oncogenic pathways at the cellular level of cancer cells and TME [38].

Consensus clustering was used to divide TCGA-CESC patients into two groups. We found that high expression of the core gene RGS1 in group 1 and low expression in group 2. DEGs between group 1 and group 2 were analyzed for functional enrichment. We found that most of the enrichment pathways were related to immunity, such as: “T cell activation”, “cytokine-cytokine receptor interaction”, and “adaptive immune response”. T cells are the focus of anti-tumor immunotherapy, especially CD8 + T cells. Because it can directly bind to the cell surface delivery of MHC I peptides to directly kill tumor cells [39]. Currently, CD4 + T cells have received more and more attention, but their function in anti-tumor immunity is bidirectional [40]. Endogenous CD4 + T cells can enhance TME immune tolerance and promote tumor growth [41]. In contrast, adoptive CD4 + T cells have been proven to have the ability to successfully elicit immune responses. In several cancers expressing MHC II, the intrinsic expression of MHC II in cancer cells is a target directly identified by CD4 + T cells and is a marker of good cancer prognosis [42]. To further grasp the extent of immune infiltration between the two groups, we used ESTIMATE, CIBERSORT, and ssGSEA, which suggested that the tumor immune response in group 1 may be more active than group 2. TME consists of more than 30 different tumor-infiltrating non-malignant cell types, as well as ECM, which both promote tumorigenesis and play key roles in anti-tumor immune responses [43]. Tumor-associated macrophages (TAMs) are the main component of TME, and have a vital function in the proliferation, invasion and metastasis of tumor cells and the inhibition of T cells anti-tumor immune response. The ability of TAMs to switch between M1 (pro-inflammatory) and M2 (pro-tumor) is a marker of poor prognosis [44]. At present, since the role of T cells in cancer immunity has been widely understood, more and more studies have been conducted on chimeric antigen receptor (CAR)-T cell therapy for cervical cancer. Unfortunately, unlike hematologic malignancies, cervical cancer is a solid tumor that may result in CAR-T cell depletion due to protection by the immune-silencing microenvironment [45]. RGS1 mediates T cell retention, leading to T cell depletion, which may be a target for improved CAR-T therapy in cervical cancer. In the expression analysis of immune checkpoint targets, we found group 1 had higher expression than group 2, indicating that patients in group 1 may have a better response to ICIs therapy.

Next, we studied the effect of RGS1 on the clinical prognosis of TCGA-CESC patients. We found that although RGS1 is considered to be a tumor promoter, the level of RGS1 expression was low in late FIGO stage, late T stage and M1 stage. In the analysis of DSS and PFI, patients in the "Dead" state had lower RGS1 expression than those in the "Alive" state. This reverse expression may be due to the fact that RGS1 is mainly expressed in B lymphocytes, T lymphocytes and other immune cells in the TME. In patients with advanced cancer or "Dead" survival, the immune cells were depleted and infiltrated less, leading to lower expression of RGS1.

Finally, we analyzed the expression and biological function of RGS1 in cervical cancer by experiments. qRT-PCR and immunohistochemistry tests of the patient tissues confirmed that the expression of RGS1 in cancerous tissue was higher compared to normal tissue, while the expression of squamous cell carcinoma in cancerous tissue was highest. Some recent studies have found that about 5% of cervical squamous cell carcinoma and 15–25% of cervical adenocarcinomas are HPV negative [46, 47]. Although more than 80% of cervical cancer is associated with HPV infection, HPV infection positive is one of the factors for a good prognosis of cervical cancer [48]. The study of Yu et al. speculated that the influence of HPV infection on the prognosis of patients with cervical cancer might be due to the increased activity of immune cells and differences in metabolic pathways [49]. Our study found that RGS1 protein expression was higher in HPV-E6-positive patients than HPV-E6-negative patients in both cervical squamous cell carcinoma and adenocarcinoma. It is further speculated that HPV infection may increase RGS1 protein expression and activate more immune cells, but whether HPV infection directly regulates RGS1 still needs more experimental verification. Zhang et al. and Wang et al. have demonstrated that RGS1 can promote tumor cell invasion and migration in osteosarcoma and melanin [33, 50]. Using cervical cancer cell lines, we demonstrated that knockdown of RGS1 inhibited the proliferation, invasion, migration and promoted apoptosis of cancer cells. In vivo experiments with a focus on nude mice tumorigenesis revealed that, RGS1 knockdown notably inhibited the growth of cervical cancer transplanted tumors. This study once again verified the tumor-promoting effect of RGS1 in cervical cancer.

## Conclusions

Using WGCNA analysis and differential gene analysis of FIGO stage, we found that the gene RGS1 that can affect the immune microenvironment and FIGO stage in TCGA-CESC patients. Subsequently, 10 key genes interacting with RGS1 were identified using PPI network, and their functional and immune correlations were analyzed. By clustering TCGA-CESC patients based on the expression of RGS1 and key genes, it was found that group 1 (RGS1: high expression) had more immune cell penetration and the proportion of ICI targets than group 2 (RGS1: low expression), indicating that group 1 had a better response to immunotherapy. It was proved that RGS1 was highly expressed in cervical cancer tissues, especially in HPV-E6 positive cancer tissues and accelerated the malignant development of cervical cancer in vivo and in vitro experiments. Our results demonstrated the potential of RGS1 as a target for cervical cancer immunotherapy and added new possibilities for immunotherapy in patients with cervical cancer.

## Data Availability

The datasets used and/or analysed during the current study are available from the corresponding author on reasonable request.

## Abbreviations

DEGs: Differentially expressed genes
ICIs: Immune checkpoint inhibitors
PFS: Progression-free survival
OS: Overall survival
RGS: Regulators of G-protein signaling
GPCRs: G protein-coupled receptors
TCGA: The cancer genome atlas
TOM: Topological overlap matrix
GS: Gene significance
MM: Module membership
GO: Gene ontology
TME: Tumor microenvironment
BP: Biological process
MF: Molecular function
CC: Cellular component
KEGG: Kyoto encyclopedia of genes and genomes
GSEA: Gene set enrichment analysis
DAPI: 4’, 6-Diamino-2-phenylindoles
CTL: Cytotoxic T lymphocytes
GAP: GTPase-activating protein
MDSCs: Myeloid derived suppressor cells
ECM: Extracellular matrix
TAMs: Tumor-associated macrophages
